# Regulating hippocampal hyperexcitability through GABAB Receptors

**DOI:** 10.1002/phy2.278

**Published:** 2014-04-23

**Authors:** Min Lang, Homeira Moradi‐Chameh, Tariq Zahid, Jonathan Gane, Chiping Wu, Taufik Valiante, Liang Zhang

**Affiliations:** 1 Toronto Western Research Institute, University Health Network, Toronto, Ontario, Canada; 2 Department of Physiology, University of Tarbiat Modares, Tehran, Iran; 3 Department of Surgery (Division of Neurosurgery), University of Toronto, Toronto, Ontario, Canada; 4 Department of Medicine (Division of Neurology), University of Toronto, Toronto, Ontario, Canada

**Keywords:** Allosteric, EEG, epilepsy, hippocampus, kindling, mice, seizure, slices

## Abstract

Disturbances of GABAergic inhibition are a major cause of epileptic seizures. GABA exerts its actions via ionotropic GABAA receptors and metabotropic G protein‐coupled GABAB receptors. Malfunction of GABAA inhibition has long been recognized in seizure genesis but the role of GABAB receptors in controlling seizure activity is still not well understood. Here, we examined the anticonvulsive, or inhibitory effects, of GABAB receptors in a mouse model of hippocampal kindling as well as mouse hippocampal slices through the use of GS 39783, a positive allosteric GABAB receptor modulator, and CGP 55845, a selective GABAB receptor antagonist. When administered via intraperitoneal injections in kindled mice, GS 39783 (5 mg/kg) did not attenuate hippocampal EEG discharges, but did reduce aberrant hippocampal spikes, whereas CGP 55845 (10 mg/kg) prolonged hippocampal discharges and increased spike incidences. When examined in hippocampal slices, neither GS 39783 at 5 *μ*mol/L nor the GABAB receptor agonist baclofen at 0.1 *μ*mol/L alone significantly altered repetitive excitatory field potentials, but GS 39783 and baclofen together reversibly abolished these field potentials. In contrast, CGP 55845 at 1 *μ*mol/L facilitated induction and incidence of these field potentials. In addition, CGP 55845 attenuated the paired pulse depression of CA3 population spikes and increased the frequency of EPSCs in individual CA3 pyramidal neurons. Collectively, these data suggest that GABABB receptors regulate hippocampal hyperexcitability by inhibiting CA3 glutamatergic synapses. We postulate that positive allosteric modulation of GABAB receptors may be effective in reducing seizure‐related hyperexcitability.

## Abstract (precis)

GABAB positive modulator GS 39783 attenuated, whereas GABAB antagonist CGP55845 facilitated hippocampal EEG spikes in kindled mice and excitatory field potentials in hippocampal slices. We postulate that GABAB receptors may inhibit CA3 glutamate synapses and hence regulate hippocampal hyperexcitability.

## Introduction

GABA is the main inhibitory neurotransmitter in the mammalian central nervous system. GABA binds to two receptors subtypes: ionotropic GABA receptors and metabotropic G‐protein‐coupled GABAB receptors. Activation of GABAA receptors produces Cl^−^‐dependent synaptic inhibition (Olsen and Sieghart 2009), whereas activation of GABAB receptors induces K^+^‐dependent hyperpolarization, or slow IPSPs, as well as inhibits voltage‐gated calcium currents and hence transmitter release of both glutamatergic and GABAergic synapses (Deisz and Lux 1985; Gähwiler and Brown 1985; Thompson and Gähwiler 1992; Bowery 2010; Pinard et al. 2010; Chalifoux and Carter 2011; Gassmann and Bettler 2012). As such, GABAB receptors are thought to play an important role in regulating physiological and pathological brain network activities (Kohl and Paulsen 2010; Groen 2011).

Disturbances of GABAergic inhibition are considered a major cause of epileptic seizures (Ben‐Ari and Holmes 2005). A loss of GABAergic inhibitory interneurons (Maglóczky and Freund 2005; Tóth et al. 2010) and malfunction of GABAA receptor‐mediated inhibition (Ferando and Mody 2012; Joshi et al. 2013; Löscher et al. 2013) have long been recognized in brain tissues of epileptic patients and relevant animal models. Alterations in GABAB receptors have also been implicated in seizure activities. mRNA expression and immunoreactivity of GABAB receptors (Muñoz et al. 2002; Gambardella et al. 2003; Princivalle et al. 2003), as well as GABAB‐mediated pre‐ and postsynaptic responses (D'Antuono et al. 2004; Teichgräber et al. 2009) are decreased in discrete cortical/hippocampal areas of epileptic patients compared to nonepileptic controls. Decreases or alterations in mRNA expression and immunoreactivity of GABAB receptors (Francis et al. 2002; Furtinger et al. 2003; Straessle et al. 2003) and GABAB‐mediated responses (Asprodini et al. 1992; Mangan and Lothman 1996; Wu and Leung 1997; Chandler et al. 2003; Gloveli et al. 2003; Leung and Shen 2006; Qu et al. 2010) are also recognized in animal models of epileptic seizures. In addition, transgenic mice with deficient GABAB receptors exhibit generalized seizure activities (Schuler et al. 2001; Vienne et al. 2010). Furthermore, the application of GABAB receptor antagonists can induce or exacerbate seizure activities (Karlsson et al. 1992; Badran et al. 1997; Vergnes et al. 1997; Carai et al. 2002; Kantrowitz et al. 2005; Leung et al. 2005; Tsai et al. 2008; but see Han et al. 2012 for absence seizure models), whereas application of the GABAB receptor agonist baclofen attenuates seizure activities (Sperber et al. 1989; Karlsson et al. 1992; Wurpel 1994; Dugladze et al. 2013). However, baclofen applications can also promote seizure or epileptiform activities (Karlsson et al. 1992; Watts and Jefferys 1993; Sutor and Luhmann 1998; Dugladze et al. 2013) largely by inhibiting GABAergic transmission (Dugladze et al. 2013).

Recent studies report that allosteric positive modulators of GABAB receptors offer anticonvulsive actions in animal models (Pacey et al. 2011; Mareš 2012; Mareš et al. 2013). When administered via intraperitoneal or subcutaneous injections, these modulators attenuated motor seizures induced by either auditory stimulation or pentylenetetrazole, as well as cortical EEG discharges induced by local electrical stimulation. These findings are of great interest as these allosteric modulators are thought to target only activated GABAB receptors and have fewer side effects than full agonists (Adams and Lawrence 2007; Pin and Prézeau 2007; Froestl 2010). However, the above anticonvulsive observations were made largely in young animals with acute seizures. The issue still remains as to whether GABAB allosteric positive modulators offer anticonvulsive or inhibitory actions in adult animals with chronic seizures, and if so, how these modulators affect the targeted local brain circuitry. We explore these issues in this study using a mouse model of hippocampal kindling and mouse hippocampal slices in vitro.

## Methods

### Animals

Male C57 black mice (Charles River Laboratory, Quebec, Canada) were used. Kindling experiments were conducted in adult mice (initial ages 6–9 months), and brain slices were prepared from 1‐ to 3‐month‐old naïve mice. These animals were housed in a vivarium that was maintained at 22°C with a 12:12 h light and dark cycle. All experimental procedures described in this study were reviewed and approved by the University Health Network Animal Care Committee in accordance with the guidelines of Canadian Council on Animal Care.

### In vivo experiments

#### Surgery and electrode implantation

Mice were operated under isoflurane (2%) anesthesia. Bipolar electrodes were placed bilaterally in the CA3 area (bregma −2.5 mm, lateral 1.3 mm, and depth 3.0 mm; Franklin and Paxinos 1997) and a reference electrode was placed into the right frontal lobe area (bregma +1 mm, lateral 2.0 mm, and depth 0.5 mm). These electrodes were secured onto the skull via a modified glue‐based method (Jeffrey et al. 2013). All electrodes were made with polyamide‐insulated stainless steel wires (outer diameter 0.2 mm; Plastics One, Ranoake, VA). The locations of implanted electrodes were verified by hippocampus‐specific EEG signals as well as histological assessment as we previously described (Jeffrey et al. 2013, 2014).

#### Kindling process

After the electrode implantation, the animals were allowed to recover for at least 7 days before further experimentation. Mice were kindled through unilateral CA3 stimulations via a train of repetitive stimuli (60 Hz for 2 sec, constant current pulses with a duration of 0.5 ms and intensities of 20–150 *μ*A; Jeffrey et al. 2013). The stimuli were generated by a Grass stimulator and delivered through an isolation unit (model S88, Natus Neurology Incorporated, Warwick, RI). Kindling stimulation was applied daily until an afterdischarge (AD) event of ≥5 sec was elicited. An ascending series of stimuli were then used to determine the AD threshold for each animal. In the ascending series, stimuli were applied from 10 to 150 *μ*A (10 *μ*A per increment) with 5 min breaks. The stimulus intensity at which an AD event of ≥5 sec was elicited was considered the AD threshold. Mice were then stimulated daily at 125% of their AD threshold and were considered fully kindled when five‐consecutive stage 5 seizures were elicited. Once fully kindled, the ascending series was applied again to each mouse to determine the final AD threshold. All drug tests were conducted at 125% of the final AD threshold. Fully kindled mice were stimulated at 125% of the final AD threshold on nontesting days to ensure the stability of ADs and motor seizures.

Motor seizures were scored using a modified Racine scale for the mouse (Reddy and Rogawski 2010). Briefly, stage 0 – no evident changes in behavioral response; stage 1 – chewing or head nodding; stage 2 – more chewing and head nodding; stage 3 – single or bilateral forelimb clonus; stage 4 – bilateral forelimb clonus and rearing; and stage 5 – loss of righting reflex. Behavioral responses were recorded using a Logitech high definition camera and were scored by experimenters blinded to experimental conditions.

#### EEG recording and analysis

Recordings were made using a microelectrode AC amplifier (model 1800, AM Systems, Carlsberg, WA). Signals were collected in a frequency bandwidth of 0.1–1000 Hz, amplified 1000× and then digitized at 5000 Hz (Digidata 1300; Molecular Device/Axon Instruments, Weatherfor, TX). Data acquisition, storage, and analysis were done using PClamp software (version 10; Molecular Device/Axon Instruments).

Hippocampal ADs were recorded from the CA3 contralateral to the stimulation site. The ADs were recognized as repetitive single or poly‐spike waveforms that displayed amplitude at least two times above background signals and durations of ≥5 sec. Spontaneous EEG spikes were recorded from the stimulated CA3 site. These spikes were recognized as intermittent events with amplitudes of ≥6 times the standard deviation above background signals and base durations of ≥30 ms (El‐Hayek et al. 2013). The event detection function (threshold search method) of the PClamp software was used to detect EEG spikes. Detected events were then visually inspected and false events were rejected before further analysis.

#### Drug treatments

GS 39783 and CGP 55845 (Tocris, Bristol, UK) were initially dissolved in DMSO as stock solutions and then diluted in saline for intraperitoneal injections. The final doses of applied DMSO were ≤500 *μ*L per kg body weight. Saline injections alone were used as controls. To examine drug effects on evoked ADs and motor seizures, GS 39783 (5 mg/kg) or CGP 55845 (10 mg/kg) were injected 15 min before the kindling stimulation. To examine the effect of these drugs on spontaneous EEG spikes, GS 39783 or CGP 55845 at the above dose was injected 4 h after the most recent kindling stimulation and EEG signals were recorded continuously for 2–3 h post injection. We used this protocol to minimize the influence of preceding ADs on spike incidences (Leung 1988, 1990; Jeffrey et al. 2013). Spike incidences were calculated from stable EEG segments of ≥30 min while the animals were immobile or asleep because these spikes occurred primarily during these inactive behaviors (Leung 1988, 1990; Jeffrey et al. 2013). Individual animals received one drug injection per test day and were given 3 days between injections to allow for sufficient recovery.

### In vitro experiments

#### Brain slices preparation

Conventional (0.5 mm thickness) or thick (0.7–1 mm thickness) hippocampal slices were prepared as described previously (Wu et al. 2005a,b; El‐Hayek et al. 2013). Briefly, the animals were anesthetized by an intraperitoneal injection of sodium pentobarbital (70 mg/Kg; Somnotol, WTC Pharmaceuticals, Cambridge, Ontario, Canada) and transcardiacally infused with cold (4°C) artificial cerebrospinal fluid (ACSF) before decapitation. To prepare the thick hippocampal slices, the brain was hemi‐sectioned, brainstem‐thalamus tissues were removed, and the CA1 and dentate gyrus areas were separated along the hippocampal fissure via a glass probe (Wu et al. 2005a,b). This separation allows sufficient oxygenation during in vitro perfusion but does not interrupt functional connections of hippocampal subfields (Wu et al. 2005a,b). The brain tissue was then glued onto an agar block and transverse hippocampal slices were obtained using a vibratom. The conventional slices were sectioned horizontally from the whole brain. After the vibratom sectioning, the slices were maintained in oxygenated (95%O_2_–5%CO_2_) ACSF for 1–6 h before recordings. The ACSF contained (in mmol/L): NaCl 125, KCl 3.5, NaH_2_PO_4_ 1.25, NaHCO_3_ 25, CaCl_2_ 2, MgSO_4_ 1.3 and glucose 10 (pH of 7.4 when aerated with 95%O_2_–5%CO_2_).

#### In vitro recordings

Extracellular and whole‐cell recordings were done in a submerged chamber and at a perfusate temperature of 36°C. The slice was perfused with oxygenated (95%O_2_–5%CO_2_) ACSF at a high rate (15 mL/min), and both the top and bottom surfaces of the slice were exposed to the perfusate. Humidified gas of 95%O_2_–5%CO_2_ was allowed to pass over the perfusate to increase local oxygen tension. Previous studies, including those from our laboratory, have shown that a fast, top and bottom perfusion of the slice is important for maintaining spontaneous population activities under submerged conditions (Wu et al. 2005a,b; Hájos and Mody 2009; Hájos et al. 2009; El‐Hayek et al. 2013).

Recording electrodes were made with thin wall glass tubes (World Precision Instruments, Sarasota, FL). Extracellular electrodes were filled with a solution containing 150 mmol/L NaCl and 2 mmol/L HEPES (pH 7.4; resistance 1~2 MΩ). Patch recording electrodes were filled with a solution containing 140 mmol/L potassium gluconate, 10 mmol/L KCl, 2 mmol/L HEPES, and 0.1 mmol/L EGTA (pH 7.25; resistance 4–5 MΩ). Extracellular and single cell signals were recorded in a frequency bandwidth of 0–5000 Hz using a dual channel amplifier (700A or 700B; Molecular Devices/Axon Instruments) and digitized at 50,000 Hz. Data acquisition and storage were done using the PClamp package as described above.

#### Afferent stimulation

A bipolar electrode, made of polyimide‐insulated stainless steel wire, was placed in the CA3 striatum oriens area for stimulating the CA3 circuitry. Constant current pulses with durations of 0.1 ms were generated by the Grass stimulator and delivered every 30 sec through the isolation unit as described above. CA1 field EPSPs and paired pulse facilitation were evoked by paired stimuli at a low intensity (45–70 *μ*A) with an interpulse interval of 50 ms. CA3 population spikes and paired pulse depression (PPD) were evoked by paired stimuli at the maximal intensity (150 *μ*A) with an interpulse interval of 250 ms. Strong stimuli were used in the latter protocol because slow IPSCs sensitive to blockade by CGP 55845 were reliably induced by single stimulation at the maximal intensity (see below). The interpulse interval of 250 ms was modified from previous studies of GABAB‐mediated depression of hippocampal glutamatergic responses (Davies et al. 1993; Isaacson et al. 1993; Leung et al. 2008). To induce repetitive field potentials in conventional slices, we used a train of high‐frequency stimuli at the maximal intensity (80 Hz for 1 sec, current pulses of 150 *μ*A).

#### Data analysis

“Spontaneous” population or single cell activities were measured from data segments of 1–3 min that were collected from individual slices or neurons before (baseline controls) and at the end of a given pharmacological manipulation. Data were then pooled together for a group of slices or neurons and baseline controls were compared against measurements postdrug treatment. Spontaneous field potentials (SFPs) were detected via the event detection function of the PClamp software and detected events were visually confirmed. SFP incidences and half‐widths were calculated from ≥8 events recorded from individual slices before or at the end of drug application. Evoked field potentials were averaged from five consecutive responses that were collected before or at the end of drug application, and measurements were made from the averaged traces. The amplitude ratio of the second versus the first response was calculated to determine the magnitude of paired pulse facilitating or depression. “Spontaneous” EPSCs were analyzed from 1‐min data segments that were collected from individual neurons before and at the end of drug application. EPSCs with amplitudes of ≥10 pA, onset time (baseline‐to‐peak) of ≤10 ms and decay time of ≤35 ms were detected using the Mini Analysis Program (version 6.07; Synaptosoft, Decatur, GA), and detected events were visually verified with the false events being rejected before further analyses. Slow IPSCs were measured from three consecutive responses before and at the end of drug application. The amplitudes of slow IPSCs were measured 150 ms poststimulation artifact.

#### Drug applications

Baclofen (Sigma–Aldrich, Mississauga, Ontario, Canada) was initially dissolved in alcohol as a stock solution and then diluted ≥3000× in the ACSF. The stock solution for NBQX (2,3‐dihydroxy‐6‐nitro‐7‐sulfamoyl‐benzo(F) quinoxaline, Sigma–Aldrich) was made with distilled water. GS 39783 and CGP 55845 were initially dissolved in DMSO as stock solutions and then diluted ≥1000× in the ACSF. CGP 55845 was applied at 1 *μ*mol/L as it consistently affected the SFPs and slow IPSCs at this concentration. GS 39783 was applied at 5 *μ*mol/L because precipitations were noticed when it was added into the ACSF at higher concentrations.

### Statistics

Statistical analyses were performed using Sigmaplot software (Systat Software Inc, San Jose, CA). Student's *t*‐test or Mann–Whitney rank sum test was used for comparing baseline controls and measurements postdrug treatment. Fisher's exact test was used for comparing the propensities of SFP induction in two groups of slices. Statistical significance was set as *P* < 0.05.

## Results

### Pharmacological manipulations of GABAB receptors in hippocampal‐kindled mice

**Figure 1.**
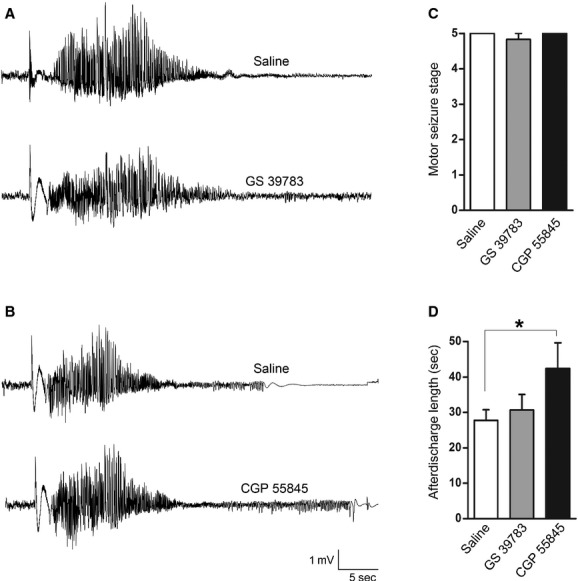
Effects of GABAB receptor modulators on hippocampal afterdischarges and motor seizures in kindled mice. (A–B) hippocampal EEG traces collected from two kindled mice, showing evoked afterdischarges following intraperitoneal injections of saline, GS 39783 (5 mg/kg, A) or CGP 55845 (10 mg/kg, B), respectively. (C–D) motor seizure scores and hippocampal afterdischarge lengths measured from five kindled mice following injections of saline, GS 39783 at 5 mg/kg or CGP 55845 at 10 mg/kg. *P < 0.05, paired *t*‐test. Note that the CGP 55845 injections prolonged hippocampal afterdischarges.

We first examined the effects of GS 39783 and CGP 55845 on evoked hippocampal afterdischarges (ADs) and motor seizures. GS 39783 is a positive allosteric modulator and CGP 55845 is a selective antagonist of GABAB receptors, respectively (Pin and Prézeau 2007; Froestl 2010). These two agents were administered via intraperitoneal injections 15 min before the hippocampal stimulation. Individual animals received one drug injection per test day and 3 days apart between injections to allow for sufficient recovery between tests. Injections of GS 39783 at 5 mg/kg did not attenuate evoked seizure activity, as hippocampal AD lengths and motor seizure stages following the GS 39783 injections were not significantly different from those measured following saline injections (*n* = 5; Fig. 1A, C–D). Injections of CGP 55845 at 10 mg/kg significantly prolonged hippocampal ADs, and AD lengths were 27.8 ± 3.0 sec and 42.4 ± 7.2 sec following the saline and CGP 55845 injections, respectively (*P* < 0.05, *n* = 5; Fig. 1B, C–D). Stage 5 motor seizures were induced following the CGP 55845 treatment, which were at the ceiling level of the seizure severity score.

**Figure 2.**
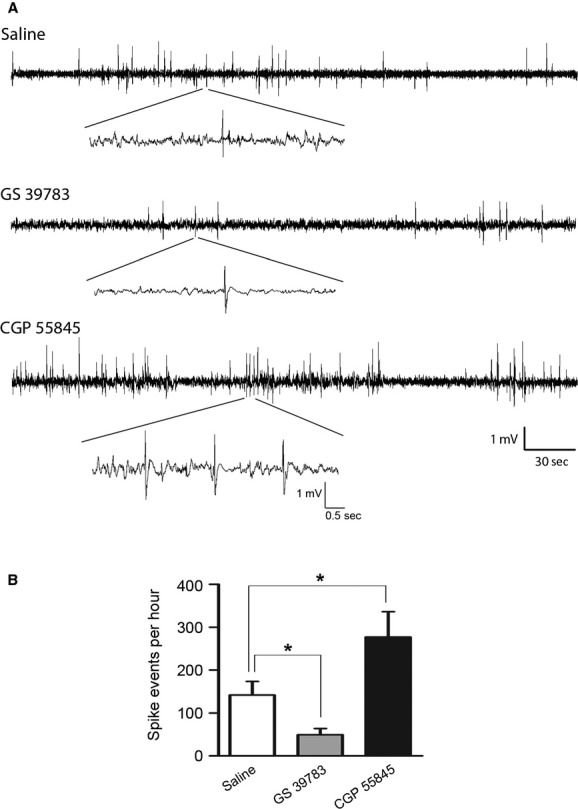
Effects of GABAB modulators on hippocampal EEG spikes in kindled mice. (A) EEG traces collected from a kindled mouse following intraperitoneal injections of saline (top), GS 39783 (5 mg/kg, middle) or CGP 55845 (10 mg/kg, bottom), respectively. The two drug injections were 3 days apart. Representative spikes are expanded. (B–C) spike incidences measured from five kindled mice following injections of saline, GS 39783 at 5 mg/kg or CGP 55845 at 10 mg/kg. *P < 0.05, paired *t*‐test. Note that GS 39783 reduced, whereas CGP 55845 increased, spike incidences.

We next examined the effects of GS 39783 and CGP 55845 on aberrant hippocampal EEG spikes in the same kindled mice. These spikes are thought to signify a state of hippocampal hyperexcitability as they rarely occur in naïve or prekindling animals and persist for several days following the kindling stimulation (Gotman 1984; Leung 1988, 1990; Morimoto et al. 2004; Jeffrey et al. 2013; see Discussion). To minimize potential influences of preceding ADs/motor seizures on hippocampal spike activity, intraperitoneal injections of saline, GS 39783 or CGP 55845 were made 4 h after the most recent kindling stimulation, and EEG signals were continuously recorded for 2–3 h post injections. GS 39783 at 5 mg/kg significantly attenuated hippocampal spike activity, and spike incidences were 142 ± 31 events/h and 49 ± 15 events/h following the saline and GS injections, respectively (*P* < 0.05, *n* = 5; Fig. 2A, B). In contrast, CGP 55845 at 10 mg/kg led to more frequent spikes (277 ± 54 events/h) compared to the saline control (*n* = 5, *P* = 0.03; Fig. 2A, B).

The rodent hippocampus exhibits irregular EEG activity in the delta (1–4 Hz) frequency band during immobility or sleep (Leung 1985; Buzsáki 1986; Buzsáki et al. 2003). The irregular activity is intermingled with brief periods of rhythmic activity in the theta band (5–12 Hz; Sainsbury 1998; Leung and Shen 2007). The latter is referred to as type‐2 or immobility theta rhythm and thought to be associated with a light level of arousal/alertness (Sainsbury 1998). The kindled mouse hippocampus also exhibited the irregular activity. Treatments with GS 39783 or CGP 55845 caused no substantial alterations in the irregular activity. The main frequency of the irregular activity, as determined by spectral analysis, was 2.6 ± 0.1 Hz and 2.6 ± 0.17 Hz following the GS 39783 and CGP 55845 injections (*n* = 5), respectively. The corresponding total power of the irregular activity after GS 39783 or CGP 55845 treatments was 381 ± 137 *μ*V^2^/Hz or 341 ± 86 *μ*V^2^/Hz, respectively (*n* = 5), which was not significantly different from the saline control (235 ± 107 *μ*V^2^/Hz; *P* = 0.40 or *P* = 0.49). The immobility theta rhythm was recognizable following GS 39783 and CGP 55845 injections. CGP 55845 did not significantly alter the peak frequency (6.4 ± 0.3 Hz) and power (283 ± 87 *μ*V^2^/Hz) of the theta rhythm relative to the saline controls (6.6 ± 0.4 Hz and 2.7 ± 0.9 *μ*V^2^/Hz; *P* = 0.72 and *P* = 0.93, respectively). Interestingly, GS 39783 injections caused a small but significant reduction in the peak theta frequency (5.8 ± 0.3 Hz; *P* = 0.04 compared to the saline treatment) but not the theta power (173 ± 57 *μ*V^2^/Hz; *P* = 0.49 compared to saline control).

Taken together, the above observations suggest that hippocampal ADs and aberrant EEG spikes of the kindled mice are controlled by GABAB receptor‐mediated inhibition. However, the intraperitoneal injections of GS 39783 or CGP 55845 might cause overall alterations of the brain activity including the hippocampal circuitry. We thus further examined whether pharmacological manipulations of GABAB receptors affect population and cellular activities of the isolated hippocampal circuitry in vitro.

### Pharmacological manipulations of GABAB receptors in mouse hippocampal slices in vitro

Previous works from our laboratory have shown that thick (0.7–1 mm) mouse hippocampal slices are able to generate spontaneous field potentials (SFPs) and that these SFPs arise from the CA3 circuitry and depend on AMPA glutamatergic activity (Wu et al. 2005b, 2009). We thus prepared thick hippocampal slices from naïve mice and examined the effects of GS 39783 or CGP 55845 on the SFPs. To determine the sensitivity of thick slices to the GABAB receptor agonist baclofen, we evoked CA1 field EPSPs and measured their amplitudes before and following applications of 0.1, 0.3, or 3 *μ*mol/L baclofen for 8 min. The amplitudes of CA1 field EPSPs were not significantly altered by 0.1 *μ*mol/L baclofen (0.9 ± 0.1 mV before and 0.9 ± 0.18 mV post baclofen, *n* = 11 slices; *P* = 0.999, paired *t*‐test) or 0.3 *μ*mol/L baclofen (0.7 ± 0.1 mV before and 0.5 ± 0.1 mV post baclofen, *n* = 8 slices; *P* = 0.256, paired *t*‐test), but greatly reduced by 3 *μ*mol/L baclofen (0.8 ± 0.1 mV before and 0.3 ± 0.04 mV post baclofen, *n* = 17 slices; *P* < 0.001, Mann–Whitney rank sum test). Based on these observations and a previous study that examines the effects of CGP 7930 (another GABAB positive allosteric modulator, Adams and Lawrence 2007) in rat hippocampal slices (Chen et al. 2006), we used 0.1 *μ*mol/L baclofen together with GS 39783 in subsequent experiments in an attempt to facilitate the in vitro action of GS 39783.

**Figure 3.**
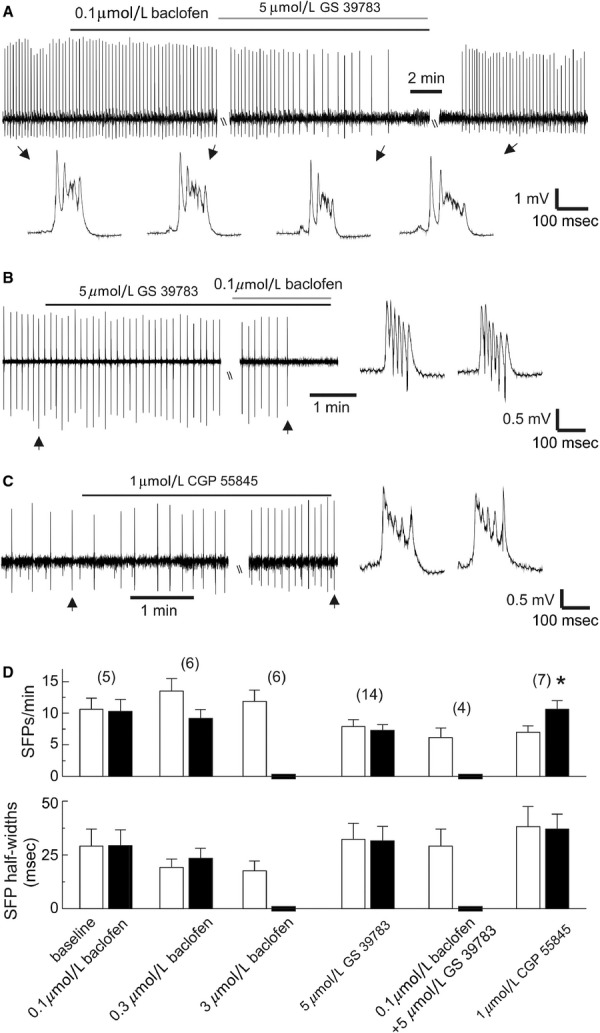
Effects of GABAB modulators on SFPs in thick mouse hippocampal slices. (A–C), SFPs recorded from the CA3 area in three slices before and following pharmacological manipulations of GABAB receptors. Lines above traces indicate application times of pharmacological agents. Arrowed SFP events are expanded. (D) Incidences and half‐widths of SFPs measured during baseline monitoring and in the presence of indicated pharmacological manipulations. Numbers of slices examined are indicated. *P = 0.031, paired *t*‐test. Note that the combined application of 5 *μ*mol/L GS 39783 and 0.1 *μ*mol/L baclofen blocked SRFs, whereas the application of 1 *μ*mol/L CGP 55845 increased SFP incidences.

Recorded from the CA3 area, the SFPs displayed amplitudes up to 5 mV, durations of 100–300 ms, and incidence rates up to 20 events per min under baseline conditions. Applications of 0.1 *μ*mol/L or 0.3 *μ*mol/L baclofen for 8 min did not significantly alter SFP incidences or half‐widths (*n* = 5 or 6 slices, *P* ≥ 0.963, Fig. 3A, D), but applications of 3 *μ*mol/L baclofen for 5–8 min blocked SFPs (*n* = 6 slices; Fig. 3D). Application of 5 *μ*mol/L GS 39783 alone for 10 min did not significantly affect SFP incidences or half‐widths (*n* = 14 slices; Fig. 3B, D). However, combined applications of 5 *μ*mol/L GS 39783 and 0.1 *μ*mol/L reversibly blocked the SFPs in four of four slices tested (Fig. 3A, B, D). In contrast, applications of 1 *μ*mol/L CGP 55845 for 8 min significantly increased SFP incidences but not SFP half‐widths (*n* = 7, *P* = 0.031, paired *t*‐test; Fig. 3C, D).

**Figure 4.**
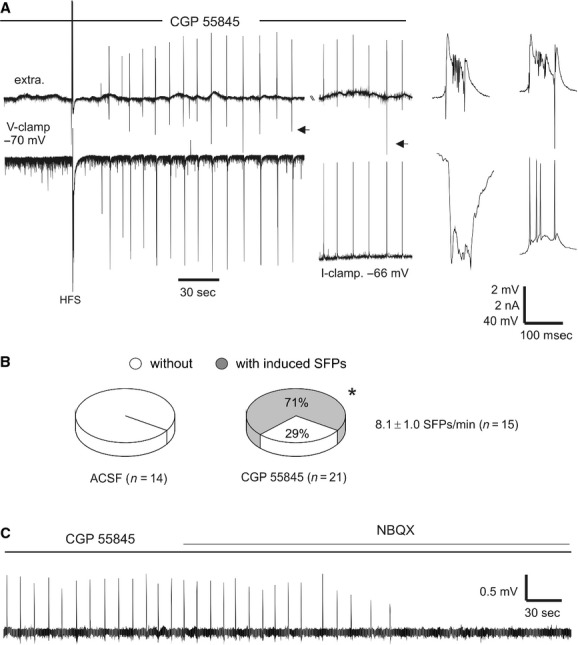
GABAB antagonist CGP 55845 facilitated SFPs in conventional hippocampal slices. (A) extra‐ and intracellular traces collected from a slice in the presence of 1 *μ*mol/L CGP 55845. A CA3 pyramidal neuron was initially voltage clamped at −70 mV and then current clamped at the indicated resting potential. Arrowed events are expanded. Note the induced SFPs and correlating intracellular activities following a train of high‐frequency stimuli (HFS, 80 Hz for 1 sec). (B) the proportion of slices with HFS‐induced SFPs in control and CGP 55845‐treated slices. *P < 0.001, Fisher's exact test. (C) induced SFPs recorded from another CGP 55845‐treated slice. Note the blockage of SFPs by 2 *μ*mol/L NBQX.

We next examined the effects of 1 *μ*mol/L CGP 55845 on SFP induction in conventional slices of naïve mice. A train of high‐frequency stimuli (80 Hz for 1 sec at the maximal intensity of 150 *μ*A) was delivered to the CA3 oriens area to induce SRFs (Fig. 4A). The high‐frequency stimulation failed to induce SFPs in 14 of 14 slices tested under baseline conditions. In contrast, of the 21 slices treated with CGP 55845, 15 slices exhibited SFPs following the high‐frequency stimulation. These induced SFPs could be continuously recorded for up to 45 min, and had an incidence of 8.1 ± 1.0 SFP events per min. Overall, the proportion of slices with induced SFPs was significantly greater following CGP 55845 treatment compared to the baseline controls (*P* < 0.001, Fig. 4B).

We conducted simultaneous extracellular and whole‐cell recordings to explore the intracellular activities underlying the induced SFPs. One example is shown in Fig. 4A, where a CA3 pyramidal neuron was recorded in the presence of 1 *μ*mol/L CGP 8845. Monitored via voltage clamp at −70 mV, this neuron exhibited frequent EPSCs before the high‐frequency stimulation and large inward currents (EPSC and spike currents) shortly following the high‐frequency stimulation. These large inward currents occurred in a close temporal relation with local extracellular SFPs. When monitored later via current clamp at resting potentials (near −66 mV), this neuron displayed large EPSCs and multiple spikes in correlation with extracellular SFPs. Similar observations were made from another three CA3 pyramidal neurons. The SFPs induced in the presence of CGP 55845 were sensitive to suppression by the AMPA receptor antagonist NBQX. Applications of NBQX at 2 *μ*mol/L for 5–6 min blocked SFPs in three of three slices tested (Fig. 4C). Collectively, these observations are in keeping with our previous studies (Wu et al. 2005b, 2009) and further suggest that the induced SFPs are mediated by strong excitatory activity in individual CA3 pyramidal neurons.

**Figure 5.**
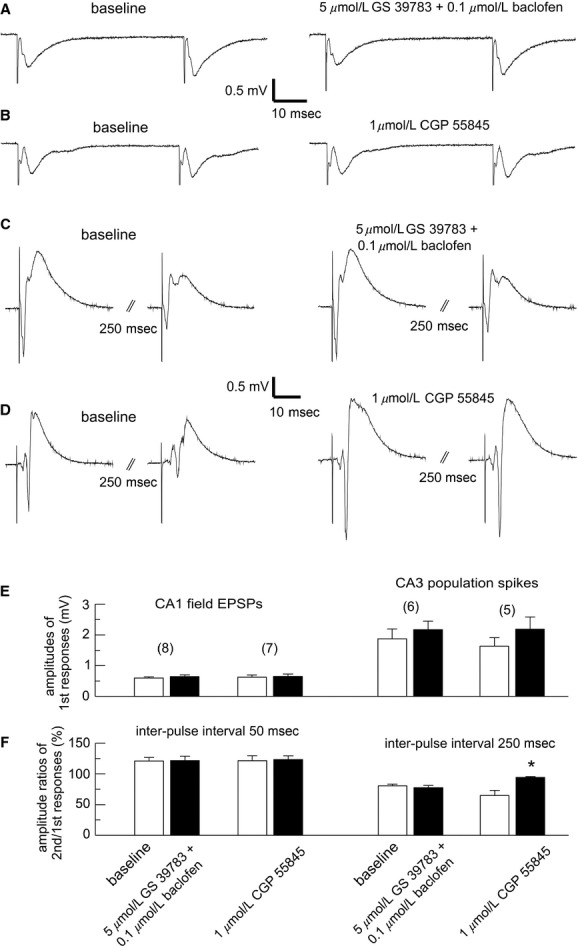
Effects of GABAB modulators on evoked field potentials in conventional hippocampal slices. (A–D) evoked potentials recorded from four slices before and following indicated pharmacological manipulations. All illustrated traces were averaged from five consecutive responses. CA1 field EPSPs (A–B) or CA3 population spikes (C–D) were evoked by paired stimuli with low (50 or 70 *μ*A) or high (150 *μ*A) intensities and interpulse intervals of 50 ms or 250 ms, respectively. (E–F), peak amplitudes of the first evoked potentials and amplitude ratios of the second versus first potentials measured from four groups of slices under baseline conditions and in the presence of indicated GABAB modulators. *P = 0.004, paired *t*‐test. Note that the paired pulse inhibition of CA3 population spikes was attenuated by CGP 55845 (D and F).

To explore the mechanisms that may underlie GABAB regulation of the SFPs, we first examined the effects of GS 39783/baclofen or CGP 55845 on evoked synaptic field potentials. These experiments were conducted in conventional hippocampal slices of naïve mice in an attempt to avoid influences of the SFPs. Paired stimuli at a low intensity and with an interpulse interval of 50 ms were used to evoke CA1 field EPSPs and paired pulse facilitation (PPF; Fig. 5A–B). Examined under baseline conditions, the mean amplitude of the 1st CA1 field EPSPs was about 0.6 mV and the mean PPF (the amplitude ratios of the second versus the first field EPSPs) was about 121%. These two parameters were minimally affected by combined applications of 5 *μ*mol/L GS 39783 and 0.1 *μ*mol/L baclofen or applications of 1 *μ*mol/L CGP 55845 for 10 min (*n* = 8 or 7 slices; Fig. 5A–B, E–F). We also evoked CA3 population spikes and paired pulse depression (PPD) via paired stimuli at a high intensity with an interpulse interval of 250 ms (Fig. 5C–D; see Methods). Under baseline conditions, the mean amplitude of the 1st CA3 population spikes was 1.9 or 1.6 mV and the mean PPD was 81.0% or 64.8% in another two groups of slices. These two parameters were not significantly altered following similar applications of 5 *μ*mol/L GS 39783 and 0.1 *μ*mol/L baclofen (*n* = 6 slices, Fig. 5C–D, E–F), but the PPD was significantly altered following applications of 1 *μ*mol/L CGP 55845 (from baseline 64.8 ± 8.3% to 94.2 ± 3.5% post CGP 55845, *n* = 5 slices; *P* = 0.004, paired *t*‐test; Fig. 5D, F).

**Figure 6.**
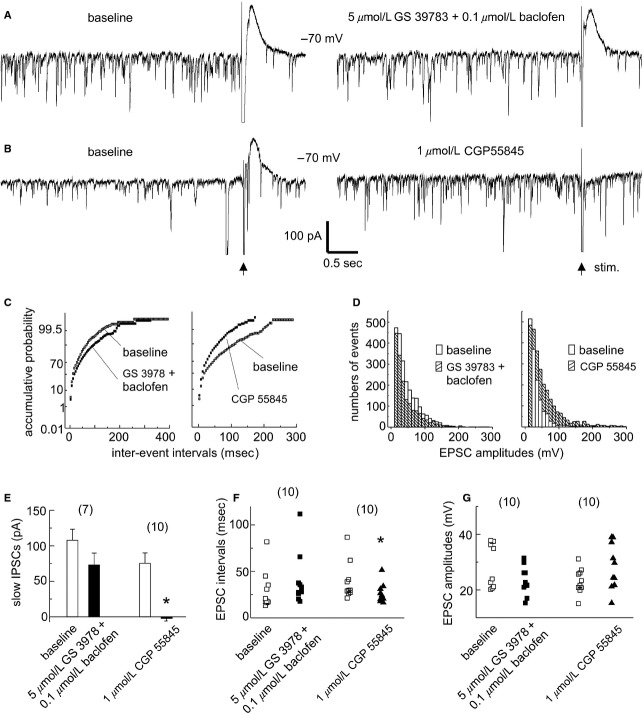
Effects of GABAB modulators on EPSCs and slow IPSCs in CA3 pyramidal neurons. (A–B) representative current traces collected from two neurons before and following indicated pharmacological manipulations. These neurons were monitored at −70 mV, and slow IPSCs were evoked by stimulating the CA3 oriens area (arrows). (C–D), EPSC interevent intervals (C) and amplitudes (D) analyzed for the above two neurons. Accumulative probability plots and amplitude histograms were generated from 1‐min data segments including the traces illustrated. E–G, slow IPSC (mean ± SE), EPSC intervals, and amplitudes measured for two groups of CA3 pyramidal neurons. Data points in F–G represent medians calculate from 1‐min data segments in individual neurons. *P ≤**0.045, paired *t*‐test.

We next examined the effects of GS 39783/baclofen or CGP 55845 in individual CA3 pyramidal neurons. These experiments were conducted in conventional hippocampal slices of naïve young adult mice. CA3 pyramidal neurons were voltage clamped at −70 mV to monitor “spontaneous” EPSCs and to minimize influences of GABAA receptor‐mediated IPSCs. Slow IPSCS were evoked every 30 sec by stimulating the CA3 oriens area (single pulse at the maximal intensity of 150 *μ*A). Under these conditions, CA3 pyramidal neurons displayed frequent EPSCs with variable amplitudes and complex waveforms and mixed synaptic currents in response to the strong afferent stimulation (Fig. 6A–B). The latter consisted of an early, large‐amplitude inward current (EPSC and spike currents) and a slow outward current (IPSC) that peaked at about 150 ms post stimulation. The interevent intervals and amplitudes of CA3 EPSCs (*n* = 10) and the amplitudes of slow IPSCs (*n* = 7) were not consistently changed following combined applications of 5 *μ*mol/L GS 39783 and 0.1 *μ*mol/L baclofen for 10–12 min (Fig. 6A, C, E–G), but inward shifts in holding currents (or depolarizing membrane potentials) were noticed (−12.3 ± 31.9 pA before and −134.4 ± 22.9 pA post GS 39783/baclofen, *n* = 10, *P* = 0.006, paired *t*‐test). Significant changes in CA3 EPSCs and slow IPSCs were observed in another 10 neurons following application of 1 *μ*mol/L CGP 55845 for 8–10 min. The interevent intervals (but not amplitudes) of CA3 EPSCs were reduced and the slow IPSCs were blocked by CGP 55845 (Fig. 6B, D, F), whereas inward shifts in holding currents were not significantly different (−46.9 ± 23.3 pA before and −76.6 ± 19.4 pA post CGP 55845, *P* = 0.088, paired *t*‐test). Taking together the effects of CGP 55845 on CA3 EPSCs and the PPD of CA3 population spikes, we suggest that the activity of CA3 glutamatergic synapses may be under inhibitory control by GABAB receptors.

## Discussion

This study was aimed to explore the role of GABAB receptors in regulating seizures and hippocampal hyperexcitability in vivo and in vitro. We used the positive allosteric modulator GS 39783 and the selective antagonist CGP 55845 (Pin and Prézeau 2007; Froestl 2010) to target endogenously activated GABAB receptors. Three main findings emerged from this study: (1) GS 39783 reduces, while CGP 55845 increases, the incidence of aberrant hippocampal spikes in kindled mice; (2) GS 39783 (together with a low concentration of baclofen) suppresses, while CGP 55845 facilitates, excitatory SFPs in hippocampal slices; and (3) CGP 55845 attenuates the PPD of CA3 population spikes and increases the frequency of CA3 EPSCs.

### Effects of GS 39783 and CGP 55845 observed from kindled mice

The kindling paradigm is widely used to model temporal lobe epilepsy and GABAB receptors are implicated in controlling the epileptogenic process of kindling models (Morimoto et al. 2004). For example, baclofen retards the development of motor seizures and increases the threshold of ADs while GABAB antagonists accelerate the development of motor seizures and increase the frequency of ADs (Karlsson et al. 1992; Wurpel 1994; Leung et al. 2005). In addition, the epileptogenic process in kindling models is associated with decreased efficacy of presynaptic GABAB inhibition on glutamatergic (Asprodini et al. 1992; Poon et al. 2006) and GABAergic synapses (Wu and Leung 1997), but enhanced postsynaptic GABAB IPSCs (Liu and Leung 2003). Our present observations, such as the prolonged ADs and increased spike incidences by CGP 55845 as well as the decreased spike incidences by GS 39783 (Figs 1 and 2), are generally in keeping with the effects of GABAB receptor ligands previously reported. However, alterations of pre‐ and postsynaptic GABAB inhibition as a result of hippocampal kindling remain to be characterized in our model.

Pacey et al. (2011) have demonstrated that intraperitoneal or subcutaneous injections of GS 39783 at 30 mg/kg attenuate auditory seizures in a mouse model of fragile X syndrome. Mareš (2012), Mareš et al. (2013) have reported that intraperitoneal injections of CGP 7930 (another GABAB‐positive allosteric modulator, Adams and Lawrence 2007) at doses of 20 and 40 mg/kg reduce seizure activities in rat models of pentylenetetrazole and cortical electrical stimulation. In contrast to these previous studies, we used GS 39783 at a low dose (5 mg/kg) in the present experiments because GS 39783 at this dose caused only slight sedative behaviors in the kindled mice. As strong sedation may influence genesis of hippocampal ADs and spikes we did not attempt to test GS 39783 at higher doses in this study.

Interictal EEG spikes manifest in patients with temporal lobe epilepsy as well as in relevant animal models and their occurrence is closely related to progression and incidences of ictal seizures (Staley et al. 2011; Avoli et al. 2013). Aberrant hippocampal EEG spikes are also recognizable in kindled animals and are thought to represent a hyperexcitable state of the kindled “epileptic” circuitry (Gotman 1984; Leung 1988, 1990; Morimoto et al. 2004). In keeping with this view, our recent study shows that hippocampal EEG spikes of kindled mice are sensitive to suppression by midazolam, a positive allosteric modulator of the GABAA receptors (Jeffrey et al. 2013). We found in the present experiments that GS 39873 at a relative low dose reduced hippocampal spike incidences in kindled mice. The spike reduction by GS 39783 was not associated with substantial alterations in the hippocampal EEG irregular activity and immobility theta rhythm. These observations suggest that positive allosteric modulation of GABAB receptors may inhibit “epileptiform” hyperexcitability while minimally affecting “physiological” EEG activity. In this context, it is of great interest and important to explore whether GABAB‐positive allosteric modulators affect the interictal spikes and recurrent seizures in other models of temporal lobe epilepsy.

### Effects of GS 39783 and CGP 55845 in hippocampal slices in vitro

Previous works from our laboratory have suggested that the SFPs are generated by the excitatory activity of the CA3 circuitry (Wu et al. 2005a, 2009). The SFPs observed in hippocampal slices are more robust compared to hippocampal EEG spikes seen in intact animals. This difference may partly reflect the activity of “disinhibited” CA3 circuitry in vitro as hippocampal EEG signals are thought to be under the control of extra‐hippocampal inhibitory inputs (Buzsáki et al. 1989). Regarding waveforms and underlying intracellular activities, the SFPs are reminiscent of interictal burst discharges previously described in conventional hippocampal slices of postischemic rats (Epsztein et al. 2006). The propensity of generating SFPs is increased in conventional hippocampal slices obtained from aged mice (El‐Hayek et al. 2013), adult mice following hypoxia‐induced seizures (Wais et al. 2009), and a mouse model of Rett syndrome (Zhang et al. 2008). Based on the above information, we consider the SFPs as hyperexcitable population activities of the CA3 circuitry in vitro.

We found that in the thick hippocampal slices, the SFPs were blocked by 5 *μ*mol/L GS 39783/0.1 *μ*mol/L baclofen and that SFP incidences were increased by 1 *μ*mol/L CGP 55845 (Fig 3). In addition, SFP induction by high‐frequency stimulation was facilitated in the conventional slices treated with 1 *μ*mol/L CGP 55845 (Fig 4). Considering that the SFPs originate from the CA3 area and are sensitive to blockade by AMPA glutamate receptor antagonists (Wu et al. 2005b, 2009; Fig 4), we speculate that GS 39783/baclofen or CGP 55845 may regulate the SFPs largely via decreasing or increasing the activity of CA3 glutamatergic synapses. Our observations that CGP 55845 attenuated the PPD of CA3 population spikes and increased the frequency of CA3 EPSCs are supportive of this view. However, GS 397835/baclofen did not consistently alter these two parameters.

Multiple factors may influence the in vitro effects of GS 39783 and CGP 55845. CGP 55845 binds to the extracellular domain of GABAB receptors with a low‐nmol/L affinity, whereas GS 39783 binds to the transmembrane domain of GABAB receptors and potentiates GABA‐stimulated GTP(*γ*s) binding at low *μ*mol/L concentrations (Pin and Prézeau 2007; Froestl 2010). We found in pilot experiments that CGP 55845 was soluble when added into the oxygenated ACSF at ≥10 *μ*mol/L, whereas GS 39783 caused precipitations when used at concentrations higher than 5 *μ*mol/L. The above differences may render GS 39783 less effective than CGP 55845 when tested in hippocampal slices. In addition, because positive allosteric modulators potentiate only activated GABAB receptors (Adams and Lawrence 2007; Pin and Prézeau 2007; Froestl 2010), robust network activities such as the SFPs may be associated with GABAB receptor activation at a higher level relative to singly evoked field potentials and thus sensitive to regulation by GS 39783/baclofen treatments. Furthermore, our assessments of CA3 EPSCs might be complicated by experimental errors. In our experiments, hippocampal slices were perfused with the ACSF at a high rate (15 mL/min) and maintained at a near‐physiological temperature (36C°) to promote spontaneous network activities under submerged conditions (Wu et al. 2005a,b; Hájos and Mody 2009; Hájos et al. 2009; El‐Hayek et al. 2013). Under these experimental conditions, it was difficult to keep stable whole‐cell recordings over a relatively long period. The inconsistent effects of GS 39783/baclofen on CA3 EPSCs might be partly due to space‐clamp limitation, changes in recording quality and/or the activity of individually recorded neurons as unexpected inward shifts in holding currents were noticed following GS 39783/baclofen treatments. Caution should also be taken when interpreting the effects of CGP 55845 on CA3 EPSCs, although the blockade of slow IPSCs by this agent is in agreement with previous studies (see review by Pinard et al. 2010).

The CA3 EPSCs we observed might largely arise from the CA3 recurrent circuitry because the CA3 pyramidal neurons interconnect intensively via their axonal collaterals (Witter 2007) and the mossy fiber‐CA3 synapses have a high failure rate when examined under standard in vitro conditions (Jaffe and Gutiérrez 2007). Previous studies have shown that baclofen is far more potent at inhibiting glutamate or GABA release (IC_50_ of 0.4–0.5 *μ*mol/L) than inducing postsynaptic outward currents or hyperpolarization (EC_50_ of 15–55 *μ*mol/L; see review by Pinard et al. 2010) and that synaptically released GABA can also effectively inhibit hippocampal glutamatergic activities (Davies et al. 1993; Isaacson et al. 1993). Based on the above information, we speculate that the increase in CA3 EPSC frequency by CGP 55845 (Fig 6) may primarily reflect a removal of GABAB‐mediated inhibition on CA3 recurrent glutamatergic synapses. However, as individual CA3 pyramidal neurons act as both pre‐ and postsynaptic neurons in the CA3 recurrent circuitry, further work is needed to isolate the pre‐ versus postsynaptic actions of GABAB antagonists and positive allosteric modulators in the CA3 circuitry.

We hypothesize that the SFPs, and perhaps also other strong hippocampal population activities, are generated by CA3 network activity involving both glutamatergic and GABAergic synapses (Beenhakker and Huguenard 2009). The latter may lead to an elevation of extracellular GABA and subsequent activation of GABAB receptors on GABAergic terminals (autoreceptors) as well as on neighboring glutamatergic terminals (heteroreceptors; Isaacson et al. 1993). The resulting GABAB inhibition of CA3 glutamatergic synapses may serve as an inhibitory mechanism that regulates SFP occurrence. In this context, GS 39783/baclofen or CGP 55845 treatments may regulate the SFPs largely via enhancing or attenuating GABAB inhibition on CA3 glutamatergic synapses. Activation of postsynaptic GABAB receptors may participate in the generation of post‐SFP hyperpolarization in CA3 pyramidal neurons (Wu et al. 2005b, 2009) thereby reducing the excitability of the CA3 recurrent circuitry. A similar scenario may explain the facilitated SFP induction by CGP 55845, as the high‐frequency stimulation may elevate extracellular GABA (Ghijsen and Zuiderwijk 2007) and subsequently activate pre‐ and postsynaptic GABAB receptors (Toprani and Durand 2013). CGP 55845 may block such poststimulation GABAB inhibition causing a build‐up of excitatory activities in the CA3 circuitry and thus increasing the propensity of SFP induction by high‐frequency stimulation.

In summary, we provide in vivo and in vitro evidence suggesting that GABAB receptors play a significant role in the regulation of hippocampal hyperexcitability. Considering that GABAB‐positive allosteric modulators offer beneficial behavioral effects without overt side effects in several animal models (Adams and Lawrence 2007; Pin and Prézeau 2007; Froestl 2010), we postulate that positive allosteric modulation of GABAB receptors may serve as a clinically relevant strategy for the management of spontaneous epileptic seizures.

## Acknowledgments

The authors thank Mr. Robert Wither for critical reading of the manuscript.

## Conflict of Interest

None declared.
